# Persistence and Degradation of Bt Toxin in Two Soil Types Under Different Sterilization Regimes

**DOI:** 10.3390/toxins18040168

**Published:** 2026-03-30

**Authors:** Yixuan Fan, Ziteng Liang, Lingli Zou, Luyao Wang, Lei Ge, Kai Zhao, Yu Sun, Peng Li

**Affiliations:** 1 Biotechnology Research Institute, Shanghai Academy of Agricultural Sciences, Shanghai 201106, China; 2 Shanghai Key Laboratory of Agricultural Genetics and Breeding, Shanghai Professional Technology Service Platform of Agricultural Biosafety Evaluation and Testing, Shanghai 201106, China; 3 College of Fisheries and Life Science, Shanghai Ocean University, Shanghai 201306, China; 4 School of Pharmacy, East China University of Science and Technology, Shanghai 200237, China; 5 School of Life Sciences and Biotechnology, Shanghai Jiao Tong University, Shanghai 201199, China; 6 Shanghai Bio-full Biotech Co., Ltd., Shanghai 201106, China

**Keywords:** Bt toxin, different sterilization regimes, dynamic equilibrium, soil organic matter, safety assessment

## Abstract

With the large-scale cultivation of transgenic *Bacillus thuringiensis* (*Bt*) crops, the Bt toxin released from Bt crops is continuously introduced into the soil. Its environmental fate represents a key indicator for assessing the ecological safety of transgenic crops. However, the persistence of Bt toxin in soil is influenced by both biotic and abiotic processes, and their respective contributions under natural conditions remain unclear. This study measured water-dissolved Bt toxin concentrations in paddy soil (PS) and red soil (RS) to compare the influence of biotic and abiotic factors on the dynamic retention of exogenous Bt toxin under different sterilization methods: no sterilization, heat sterilization (HT), and irradiation sterilization (IS). The water-dissolved Bt toxin exhibited a dynamic decrease–increase–decrease trend across all three treatments in both soil types during the 30 day experimental period. Bt toxin displayed rapid adsorption during the initial 2 h stage in RS, but subsequently showed a high desorption, whereas PS probably achieved more stable bonding through soil organic matter (SOM). Different sterilization methods significantly influenced the results by altering abiotic factors: Compared to CK, HT affected soil physicochemical properties and enhanced adsorption resilience, whereas IS caused minimal impact on the soil physicochemical properties, thereby providing a more accurate reflection of abiotic processes. And microbial, as biotic facters, also influence the reduction process of Bt toxin by participating in the adsorption–desorption–degradation equilibrium process. Therefore, we infer that over time, the concentration of water-soluble Bt proteins in the soil will tend toward zero. Additionally, the initial Bt toxin concentration influenced dynamic balance by adjusting adsorption site saturability, with more pronounced desorption reversibility at 500 ng/g concentrations. Overall, this study systematically reveals the effects of soil properties, microorganisms, and sterilization methods on Bt toxin persistence. The findings underscore the importance of selecting and justifying sterilization methods in related environmental behavior studies, while providing essential guidance for the scientific assessment of environmental risks posed by transgenic crops.

## 1. Introduction

Bt toxin is an insecticidal crystal toxin produced by *Bacillus thuringiensis* (*Bt*) during the early stages of spore formation [1,2]. In this study, we specifically investigated the 60 kDa activated fragment of the Cry1Ab toxin, a member of the three-domain Cry family. Its primary applications are twofold: first, as a highly effective biological insecticide, Bt toxin offers advantages in controlling target pests, reducing insect resistance, and ensuring environmental safety [3]; second, the *Bt* gene (*Cry1Ab*), which encodes Bt toxins, is the most widely used insect-resistant gene in transgenic crops and accounts for one of the largest cultivation areas globally [4,5]. In natural soil environments, Bt toxin concentrations are typically low, and the toxin mainly exists in a particle-bound form, which renders its detection challenging via conventional enzyme-linked immunosorbent assay (ELISA) protocols [6,7,8,9]. However, the large-scale planting of transgenic Bt crops results in the continuous release of Bt toxin into the soil through pathways including root exudates, pollen deposition, and the decomposition of plant residues [10,11]. Once introduced, these exogenous Bt toxin undergoes complex interactions with soil components and microbial communities, eventually reaching a dynamic equilibrium. These processes are governed by two principal mechanisms: one category involves abiotic processes predominantly driven by adsorption, desorption, and hydrolysis [12], while the other category includes biotic processes primarily mediated by microbial degradation [13]. Understanding the adsorption, desorption, degradation, and ultimate fate of exogenous Bt toxin in soil is essential for assessing its potential impacts on soil organisms, soil ecological functions, and nutrient transfer within food webs.

The persistence of exogenous Bt toxin in soil is dynamically influenced by multiple factors, including soil physicochemical properties and microbial community composition [5]. Although microbial degradation and soil particle-mediated adsorption–desorption act concurrently, their individual contributions to Bt toxin degradation are challenging to disentangle under natural conditions. To precisely quantify the relative roles of biotic and abiotic processes in the fate of Bt toxin, soil sterilization has become an indispensable experimental approach. Currently, heat sterilization (HT) and irradiation sterilization (IS) (e.g., gamma or electron beam irradiation) are the most commonly employed soil sterilization methods, although they differ fundamentally in terms of mechanism and collateral effects [14]. HT effectively eliminates microbial activity but may induce significant alterations in soil texture, soil organic matter (SOM), and nutrient availability due to thermal stress [15]. In contrast, IS inactivates microbes by disrupting nucleic acids through high-energy radiation, with minimal reactive oxygen species generation contributing to secondary antimicrobial effects [16]. Crucially, IS has less impact on soil physicochemical properties and better preserves the native adsorption characteristics of the soil texture. Therefore, the choice of sterilization method directly impacts the accuracy of experimental conclusions. Neglecting the differences in soil physicochemical properties caused by different sterilization methods will lead to misinterpretation of the actual effects of microorganisms, thereby affecting the scientific assessment of Bt protein’s environmental behavior.

Therefore, this experiment selected two typical soils with significantly different physicochemical properties. By examining the interaction between soil type and sterilization method, this study aimed to elucidate how different sterilization techniques influence the relative contributions of biotic and abiotic processes to the persistence of Bt toxin. The findings provide a scientific foundation for improving the accuracy of environmental fate evaluations of Bt toxins and for standardizing methods in related laboratory studies.

## 2. Results

### 2.1. Alteration in Soil Physical and Chemical Properties

**Figure 1:**
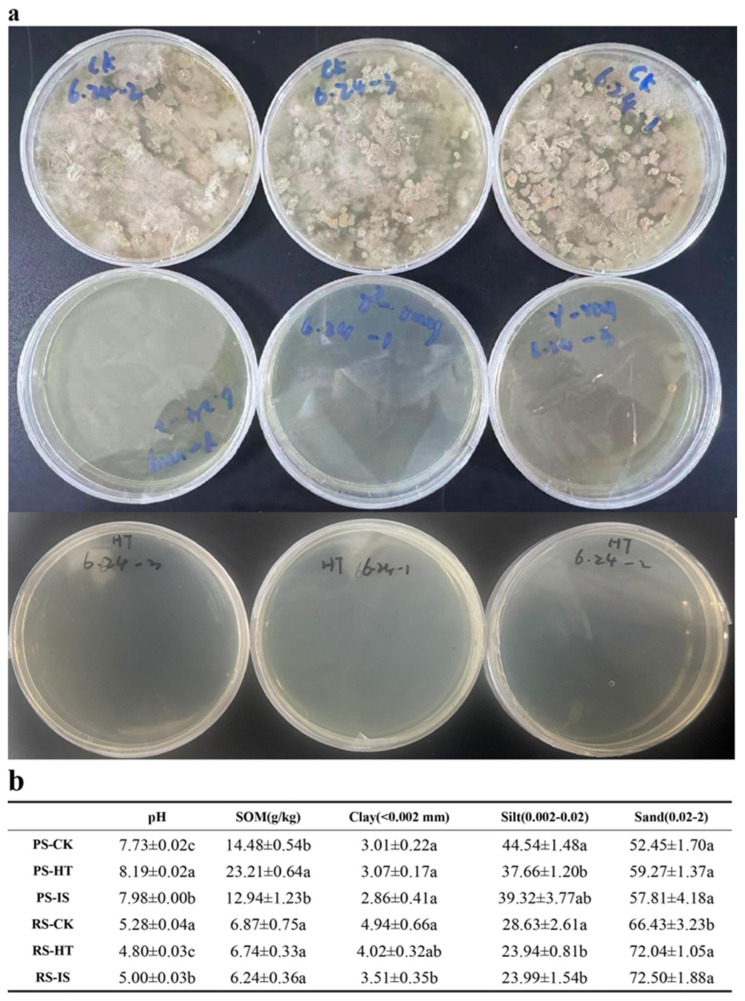
(**a**) Sterility verification by plate culture of test microbes. The three rows of plates were used to verify microorganisms derived from PS-CK, PS-IS, and PS-HT, respectively. The RS results were the same. (**b**) Physicochemical properties of soils, including three treatments for two soil types, as well as their pH, soil organic matter (SOM) content, texture, and significance (*p* < 0.05; data are presented as mean ± SD (*n* = 3)). PS: paddy soil; RS: red soil; CK: control; HT: heat sterilization; IS: irradiation sterilization (Different lowercase letters (e.g., a, b, c, ab) indicate statistically significant differences among treatments according to the LSD test or Duncan’s test at *p*< 0.05. Means sharing the same letter are not significantly different).

We found that compared with PS, RS, whether sterilized or not, was acidic. Compared with CK, RS-HT was more acidic, whereas PS-HT was more alkaline. The acidity/alkalinity of the two soil types treated using IS fell between CK and HT. In addition, the SOM content of RS was significantly lower than that of PS. Neither HT nor IS had a significant impact on the SOM content of RS. However, the SOM content in PS-HT was significantly higher than that in CK (Figure 1b, *p* < 0.05).

In terms of texture, there were no significant changes in clay and sand content in sterilized PS compared with CK. However, the content of silt in PS-HT was significantly decreased compared with both CK and IS. The texture of RS was altered following sterilization; the contents of clay and silt both decreased, although the decrease in clay content after HT was not significant. However, the sand content increased significantly post-sterilization (Figure 1b, *p* < 0.05).

### 2.2. Changes in Bt Toxin Concentration in CK Soil

**Figure 2:**
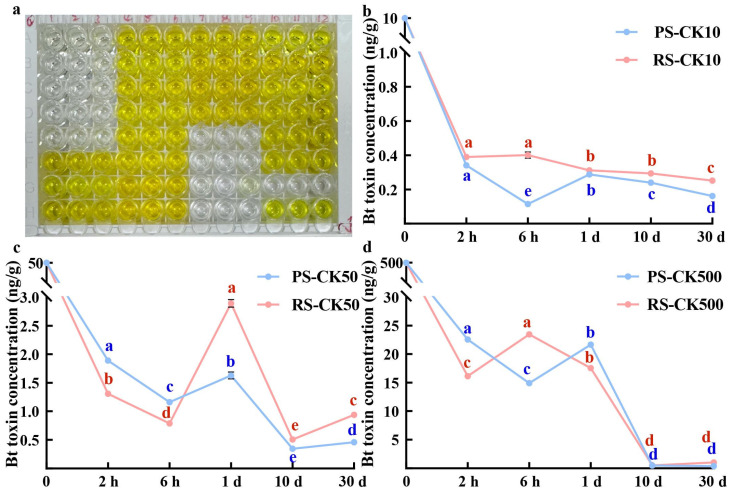
(**a**) ELISA, blank wells represent samples without added Bt toxin. Colored wells represent samples with added Bt toxin. (**b**) Toxin concentration over sampling time for PS-CK and RS-CK at an initial Bt toxin concentration of 10 ng/g. (**c**) Bt toxin concentration versus sampling time curves for PS-CK and RS-CK with an initial Bt toxin concentration of 50 ng/g. (**d**) Bt toxin concentration versus sampling time curves for PS-CK and RS-CK with an initial Bt toxin concentration of 500 ng/g. PS: paddy soil; RS: red soil (Different lowercase letters (e.g., a, b, c, d, e) indicate statistically significant differences among treatments according to the LSD test or Duncan’s test at *p*< 0.05. Means sharing the same letter are not significantly different).

The ELISA results for PS-CK indicated that, regardless of the amount of Bt toxin applied, the concentration of Bt toxin in the soil showed a trend of first decreasing, then increasing, and decreasing again over time (Figure 2a). Notably, the rate of decrease was usually the greatest during the first 6 h, after which the content of water-dissolved Bt toxin began to increase from 6 h to 1 d, followed by a decrease around 10 d, at which point the rate of decrease gradually slowed (Figure 2b–d).

However, in RS-CK, when the amount of Bt toxin applied was 50 ng/g, the content of water-dissolved Bt toxin in the soil decreased rapidly within the initial 6 h, then gradually increased from 6 h to 1 d, with subsequent decline from 1 to 30 d and ultimately equilibrium (Figure 2c). However, under the application of 10 and 500 ng/g Bt toxin, the water-dissolved Bt toxin in the soil decreased rapidly within the first 2 h, gradually rose after 2 h, and dropped again around 6 h. At this point, the rate of decrease was diminished with prolonged time and gradually tended to stabilize (Figure 2b,d).

### 2.3. Changes in Bt Toxin Concentration in Sterilized Soil

**Figure 3:**
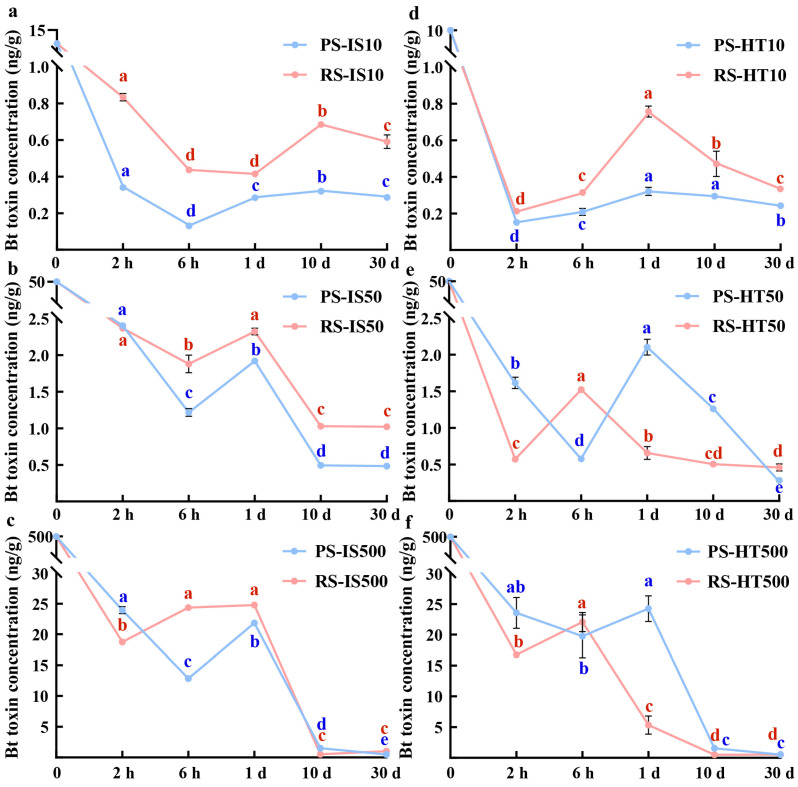
(**a**) Bt toxin concentration versus sampling time curves for PS-IS and RS-IS treated with an initial Bt toxin concentration of 10 ng/g. (**b**) Bt toxin concentration versus sampling time curves for PS-IS and RS-IS treated with an initial Bt toxin concentration of 50 ng/g. (**c**) Bt toxin concentration versus sampling time curves for PS-IS and RS-IS treated with an initial Bt toxin concentration of 500 ng/g. (**d**) Bt toxin concentration versus sampling time curves for PS-HT and RS-HT treated with an initial Bt toxin concentration of 10 ng/g. (**e**) Bt toxin concentration versus sampling time curves for PS-HT and RS-HT treated with an initial Bt toxin concentration of 50 ng/g. (**f**) Bt toxin concentration versus sampling time curves for PS-HT and RS-HT treated with an initial Bt toxin concentration of 500 ng/g. PS: paddy soil; RS: red soil; HT: heat sterilization; IS: irradiation sterilization (Different lowercase letters (e.g., a, b, c, ab, d, e, cd) indicate statistically significant differences among treatments according to the LSD test or Duncan’s test at *p*< 0.05. Means sharing the same letter are not significantly different).

The IS treatment induced a fall–rise–fall trend in the Bt toxin content of PS and RS regardless of the amount of Bt toxin applied, although small differences were detected in the rate of rise or fall for different concentrations and soil types (Figure 3a–c).

PS and RS treated with 10 ng/g and 50 ng/g of Bt toxin and PS treated with 500 ng/g of Bt toxin showed the same trend of change, with the greatest rate of decrease from 0 to 6 h, a slow increase from 6 h to 1 d, and finally a slow decline from 10 to 30 d, when the rate of decrease was significantly lower than that of the initial addition concentration (Figure 3a,b). The Bt toxin content of RS treated with 500 ng/g of Bt toxin declined rapidly from 0 h, after which the water-dissolved Bt toxin concentration began to rise at about 2 h, remained stable between 6 h and 1 d, and then declined rapidly from 1 to 10 d, with stability maintained from 10 to 30 d. The rate of decrease was significantly lower than that of the initial addition concentration (Figure 3c).

When the amount of Bt toxin applied to the PS-HT and RS-HT soils was 10 ng/g, the content of water-dissolved Bt toxin decreased rapidly within 0–2 h, then increased and decreased again after 1 d until around 30 d (Figure 3d). It is worth noting that RS-HT treated with 50 and 500 ng/g Bt toxin displayed similar trends.

When the Bt toxin content was applied to PS-HT, it was 50 and 500 ng/g; the content of the water-dissolved Bt toxin decreased rapidly from 0 to 6 h, then increased from 6 h to 1 d, and decreased again between 1 and 30 d (Figure 3e,f).

## 3. Discussion

### 3.1. Soil Type and Physicochemical Properties Are the Significant Factors Determining the Persistence of Bt Toxin

Detectable Bt toxins typically exist in a water-dissolved state, whereas Bt toxins within the soil portion are generally present in a bound state [2,17], adsorbed onto soil particles and mineral colloids. The water-dissolved, -adsorbed, or -degraded states of Bt toxins in soil constitute a dynamic, reversible process regulated by multiple factors [18]. Therefore, we speculated that the differences in physicochemical properties have a significant impact on the existence state of Bt toxins. RS is acidic and rich in iron and aluminum oxides, containing less SOM due to higher weathering levels and a lower organic carbon accumulation capacity [19], whereas PS is weakly alkaline with abundant microbial diversity and high SOM content [20]. HT reduced the microbial consumption of SOM and disrupted microbial textures, releasing various substances within them [21]. This promoted the resynthesis of SOM of organic decomposition products, resulting in a significant increase in SOM in the PS-HT soils.

RS exhibited higher clay content but lower SOM, whereas PS possessed high SOM content but significantly lower clay content than RS (Figure 1a,b). This difference in concentration was particularly evident in the initial adsorption of Bt toxin by these soils. Water-dissolved Bt toxin in RS declined more rapidly within the first 6 h, potentially due to the higher clay content and acidic environment of RS, which allowed for the rapid capture of Bt toxin during initial adsorption (Figure 2 and Figure 3). However, Bt toxin with weaker adsorption to clay particles is prone to desorption under the influence of other factors, thereby re-entering the soil as water-dissolved toxin [22]. Conversely, in PS, which has lower clay content and a weakly alkaline environment, the initial decline rate of high-concentration water-dissolved Bt toxin was slightly lower than that observed in RS. (Figure 2 and Figure 3). The higher SOM in PS could provide additional binding sites for Bt toxin, allowing it to tightly bind to functional groups on organic surfaces and resist desorption [23]. Consequently, desorption rates in PS were significantly lower compared to RS. Thus, while adsorption occurs, partially desorbed Bt toxin may be re-adsorbed by clay particles or SOM in the soil [24,25]. These two processes occur simultaneously in soil, ultimately reaching an adsorption–desorption equilibrium at a specific time.

### 3.2. Influence of Sterilization Methods and Initial Concentrations on the Persistence of Bt Toxin

**Figure 4:**
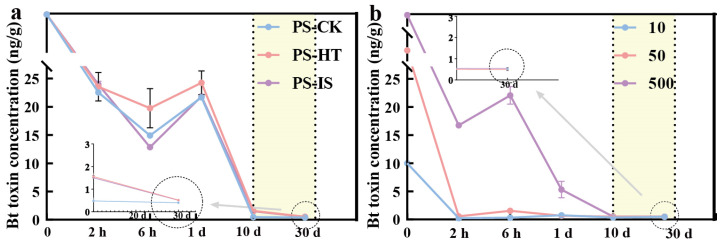
(**a**) Taking PS-500 as an example, the curves and final trends of water-dissolved Bt toxin in soil under CK, HT and IS treatments with respect to sampling time. (**b**) Taking RS-HT as an example, the curves and final trends of water-dissolved Bt toxin in soil with the addition of different concentrations of Bt toxin over sampling time. PS: paddy soil; CK: control; HT: heat sterilization; IS: irradiation sterilization.

In CK conditions, the water-dissolved Bt toxin concentration in both soils exhibited a dynamic process of rapid decline–partial increase–slow decline (Figure 4), a trend that could not be explained by adsorption–desorption. Thus, we infer that within the first 6 h following Bt toxin addition, the abundant binding sites in soil favor the influence of adsorption over microbial degradation, resulting in a rapid decrease in water-dissolved Bt toxin content [26]. As the concentration of water-dissolved Bt toxin decreases, the abundance of soil microorganisms with strong Bt toxin degradation capabilities increases [7]. During the degradation of Bt toxin, these microbes may also secrete substances such as organic acids. On the one hand, these acids assist in desorbing weakly adsorbed Bt toxin from soil; on the other hand, they compete with Bt toxin for binding sites, making it difficult for the toxin to bind. At this stage, the concentration of water-dissolved Bt toxin in the soil is reversible. Finally, the number of available adsorption sites for Bt toxin in soil gradually decreases over time, leading to concurrent reductions in both adsorption and desorption rates [27]. Simultaneously, the microbial degradation of Bt toxin continues, resulting in a gradual drop in water-dissolved Bt toxin content. Throughout this process, the concentration of water-dissolved Bt toxin in the soil achieves an equilibrium of adsorption–desorption–degradation facilitated by microbial activity.

The influence of microorganisms is primarily evident during the initial phase of Bt toxin degradation (Figure 4a). Our previous research demonstrated that Bt toxins introduced into the environment from transgenic Bt crops undergo rapid degradation, with bacteria serving as the crucial drivers of this degradation process. Furthermore, these microbial communities simultaneously enhance soil nutrient multifunctionality [1]. Moreover, the relative abundances of several genera, including *Altererythrobacter*, *Brevundimonas*, *Arthrobacter*, *Mesorhizobium*, and *Nitrospira*, were significantly correlated with Bt toxin concentrations [7]. These taxa are primarily involved in the decomposition and mineralization of proteins, as well as the metabolism of carbohydrates, nitrogen compounds, fatty acids, and amino acids in soil ecosystems. Collectively, these processes may drive shifts in microbial community composition and metabolic activities, thereby modulating the ecological effects of Bt toxins.

At identical initial Bt toxin concentrations, water-dissolved Bt toxins in both sterilized soils (HT and IS) and CK soil reached a dynamic equilibrium over time. However, due to microbial degradation, CK achieved this equilibrium point earlier than HT and IS. Compared to CK, both HT and IS methods effectively eliminated the microbial degradation of Bt toxin. However, the trend of water-dissolved Bt toxin in soil still exhibited a pattern of rapid decline–partial increase–slow decline (Figure 4a). Notably, HT and IS methods exerted differing effects on the dynamic changes in Bt toxin. For example, in PS treated with an initial Bt toxin concentration of 500 ng/g, the HT method caused a slight decrease in water-dissolved Bt toxin content between 2 h and 1 d, followed by gradual recovery to a concentration close to that at 2 h, which was slightly higher than that of the CK group. In contrast, the IS method resulted in a rapid decline in water-dissolved Bt toxin between 2 h and 1 d, followed by a slight recovery, reaching levels comparable to CK but lower than those of HT (Figure 3c,f).

Based on soil physicochemical data, it is speculated that the high temperature utilized under the HT method may have altered toxin adsorption sites and binding strength, causing Bt toxin to exhibit weaker adsorption onto SOM and soil particles. This facilitated its desorption and re-release into the water-dissolved state. In contrast, the IS had minimal impact on the soil texture, thus exerting relatively minor effects on Bt toxin dynamics in soil [28]. These results arising from sterilization methods represent variations influenced by abiotic factors, primarily driven by differences in physicochemical properties. This underscores the importance of evaluating different sterilization methods in studies examining microbial effects.

In addition to soil type and sterilization method, the initial toxin concentration also had a significant influence on the fate pathways of Bt toxin. At lower concentrations (10 ng/g), limited Bt toxin rapidly occupied irreversible adsorption sites on SOM and soil particles or underwent partial microbial degradation, resulting in a sustained decline (Figure 2b). During this process, microbial involvement in partial desorption slightly enhanced the water-dissolved Bt toxin content. But these toxins were subsequently reversed by the remaining sites, resulting in minimal fluctuation. At medium concentrations (50 ng/g), strong binding sites in soil particles and SOM became saturated after initial binding. However, some weakly adsorbed toxins desorbed due to soil physicochemical properties or microbial influences, leading to a noticeable reversibility at lower concentrations. We found that higher concentrations of Bt toxin addition can influence the activities of Bt toxin-degrading microorganisms, thereby altering soil NH_4_^+^-N, NO_2_^−^-N, and soil organic matter (SOM) contents. These changes subsequently regulate the composition of microbial functional genes and metabolites involved in carbon, nitrogen, and phosphorus cycling [1,7]. At higher concentration (500 ng/g), all binding sites in the soil rapidly became saturated with Bt toxin, with a large portion existing in a weakly adsorbed state, making desorption reversibility more readily observable. This phenomenon was more pronounced in sterilized soil. In non-sterilized soil, even after extensive adsorption, water-dissolved Bt toxin exceeded the maximum degradable capacity of soil microorganisms, requiring extended periods for degradation [29] (Figure 4b).

**Figure 5:**
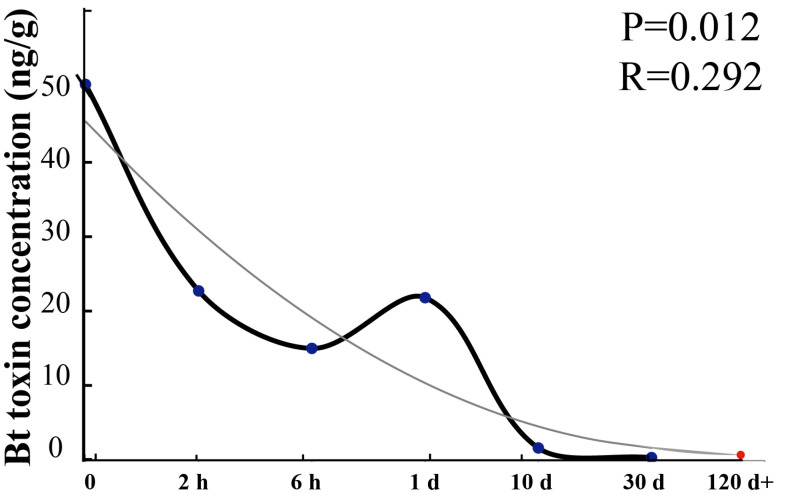
Fitting curve of water-dissolved Bt toxin concentration over time. The concentration of water-dissolved Bt toxin gradually decreased over time and approached zero after 30 days. The jagged curve represents the trend line of Bt toxin concentration changes in soil, while the smooth line indicates the predicted ultimate fate curve of water-dissolved Bt toxin in soil.

As summarized above, water-dissolved Bt toxin concentrations in both soil types, regardless of sterilization status, exhibited a consistent decrease–increase–decrease pattern of dynamic change within 30 d following the application of varying concentrations of Bt toxin. Under CK conditions, the concentration of water-dissolved Bt toxin declined rapidly during the initial phase, followed by a slight reversibility influenced by the interplay of biotic and abiotic factors. Subsequently, it gradually decreased over time through the dynamic processes of adsorption, desorption, and degradation [30]. Notably, the same temporal trend was observed even in sterilized soils, where microbial activity was eliminated, indicating that abiotic mechanisms alone could drive this characteristic. Based on these observations, and by integrating the effects of the initial Bt toxin concentration, soil physicochemical properties, microbial activity, and incubation time, we infer that following the 30 d decrease–increase–decrease phase, the concentration of water-dissolved Bt toxin will continue to decline and ultimately approach zero (Figure 5).

## 4. Conclusions

This study demonstrates that the fate of exogenous Bt toxin in soil is a dynamic process governed by multiple interacting factors. Consequently, the water-dissolved Bt toxin concentration of Bt toxin exhibits a characteristic temporal pattern of decrease–increase–decrease. Soil physicochemical properties provide the fundamental framework for this behavior: in RS, acidic conditions and high clay content facilitate the rapid initial adsorption of Bt toxin, although this binding is marked by significant reversibility; in contrast, the abundant SOM in PS promotes more stable and progressive immobilization. Moreover, microorganisms not only contribute to the biodegradation of Bt toxin but also modulate the adsorption–desorption equilibrium through metabolic activities. The initial Bt toxin concentration further influences the system via determining the degree of saturation of available adsorption sites, thereby affecting the reversibility of dynamic binding processes. Importantly, sterilization methods do not merely eliminate microbial activity but also induce abiotic alterations to the soil texture that directly affect toxin behavior. HT enhances adsorption reversibility via modifying the composition of SOM and disrupting soil texture, whereas IS exerts minimal impact on soil physicochemical properties, resulting in distinct toxin dynamics between the two methods. These results highlight the critical significance of scientifically selecting and justifying sterilization methods in environmental behavior research, while also providing an important reference for the scientific assessment of the environmental risks associated with genetically modified crops.

## 5. Materials and Methods

### 5.1. Soil Samples and Bt Toxin

The soil samples utilized in this experiment were collected from Shanghai (31°13′ N, 121°19′ E) and Yingtan (27°35′ N, 116°41′ E), Jiangxi Province. Two soil types were selected: paddy soil (PS) and red soil (RS). Soil samples were air-dried at room temperature (25 ± 2 °C) for 7 days until constant weight. Large clods were gently crushed using a porcelain mortar and pestle to break aggregates, and the material was then passed through a 20-mesh stainless steel sieve to remove stones and plant debris. The sieved soil was thoroughly homogenized by manual mixing for 10 min to ensure uniformity. The experiment was conducted following the principle of randomized grouping. Soil pH and SOM were measured for initial characterization and subsequently to assess process effects. The soil pH value was determined by the potentiometric method mentioned by He et al. [31], and the SOM content was determined by the potassium dichromate volumetric method [32].

The Bt toxin employed in this study was supplied by Beijing Zhanuosite Technology Co., Ltd. (Beijing, China)., with a stock solution concentration of 1.0 mg/g and a molecular weight of 60 kDa. The stock solution was serially diluted with distilled water to obtain the desired Bt toxin concentrations according to experimental requirements. Soil Cry1Ab toxins were quantified by enzyme-linked immunosorbent assays, as described by Dohrmann et al. (2013) [33].

### 5.2. Experimental Design and Treatments

In this experiment, PS and RS were treated using distinct sterilization methods: the non-sterilized control group (CK), the HT group, and the IS group. The Non-sterilized PS (PS-CK) soil remained untreated to preserve its natural state. The heat sterilization PS (PS-HT) soil was sterilized at 121 °C for 20 min, whereas the PS-IS soil underwent instantaneous sterilization using a 40 kGy electron beam. Before beginning the experiment, all treated soil samples were subjected to plate culture assays. The effectiveness of the sterilization process was verified by plating the samples on Luria–Bertani (LB) agar. The results of the plate coating showed that no bacterial growth occurred in either the high-temperature sterilization group or the irradiation sterilization group, confirming the sterility of the experimental soil. Sampling was also conducted in a clean bench to ensure aseptic conditions throughout the experimental process. These dual measures collectively ensured the inactivation of microbial activity in both sterilization treatments.

The three method groups for both soil types were further subdivided based on the concentrations of Bt toxin applied (0, 10, 50, and 500 ng/g). Meanwhile, soil pH and SOM were monitored as key treatment-dependent indicators. For sample preparation, 40 g of soil was weighed into a 50 mL centrifuge tube, mixed with 20 mL of distilled water containing the desired concentration of Bt toxin (obtained via 10-fold gradient dilution), and allowed to equilibrate. Each subgroup included three replicates. Sampling was performed at designated time intervals (2 h, 6 h, 1 d, 10 d, and 30 d), with 2.0 g of soil collected per replicate and stored at −20 °C. The Bt toxin concentration was quantified using a Cry1Ab/Ac ELISA detection kit (Shanghai Youlong Biotech. Co., Ltd. (Shanghai, China)) following the enzyme-linked immunosorbent assay protocol.

### 5.3. Statistical Analysis

Data were systematically processed utilizing specialized software, including IBM SPSS Statistics version 23.0, R Studio (version 4.5.0), and Origin 2024b (OriginLab Corporation, Northampton, MA, USA). All data were analyzed using IBM SPSS Statistics version 23.0. Prior to analysis, the normality of the data distribution was assessed using the Shapiro–Wilk test, and homogeneity of variances was verified using Levene’s test. For comparisons between two groups, statistical significance was determined using a two-tailed Student’s *t*-test. For comparisons involving multiple groups (e.g., different toxin concentrations or time points), one-way analysis of variance (ANOVA) was performed. When ANOVA indicated significant differences, post hoc multiple comparison tests were conducted to identify specific group differences. The post hoc test used was Tukey’s HSD test and Duncan’s test. A *p*-value of less than 0.05 was considered statistically significant.

## Data Availability

The original contributions presented in this study are included in the article. Further inquiries can be directed to the corresponding authors.
